# The deterioration of the Pueblo Bonito Great House in the Chaco Culture National Historical Park, New Mexico, USA

**DOI:** 10.1371/journal.pone.0266099

**Published:** 2022-04-05

**Authors:** Henry L. Short

**Affiliations:** U.S. Department of the Interior, Santa Fe, New Mexico, United States of America; Al Mansour University College-Baghdad-Iraq, IRAQ

## Abstract

Pueblo Bonito is the iconic pre- Columbian structure in Chaco Culture National Historical Park, a World Heritage Site in northwestern New Mexico, USA. The structure, dating to about 850–1150 Current Era, and built of quarried sandstones, wooden timbers and a mud mortar, has been the subject of archaeological investigations for over a century. The present study is based on the examination of historical photographs of Pueblo Bonito dating from 1887 to the 1920s. It is a retrospective assessment to determine if structural damages, depicted on the photographs, could be attributed to identifiable agents that might have been present at the time of Pueblo Bonito occupancy. A likely causal agent of deterioration at Pueblo Bonito was the inability of Ancestral Puebloan engineers to manage the impacts from the annual precipitation, presently measured at about 220 mm. A resulting time-dependent event was rot to wetted roof and ceiling timbers, lintels, and wall support beams which required decades of incubation by wood decay fungi to reduce wood tensile strength to levels leading to roof and wall collapse. Important time- independent events that could occur any time after construction include water action on the mud mortar which resulted in unstable gravity load paths in stone walls, ponding of water in walls which when frozen would lead to the blowout of wall segments, and the occasional flood that disrupted foundations. Pueblo Bonito may have been an occupation site for centuries but the lifetime of individually constructed rooms may have only been decades, resulting in several build- repair- or abandon cycles being part of the history of that Great House.

## Introduction

This is a story of a pre-Columbian Native American population who for three or four hundred years harnessed a form of potential energy and of the natural forces that over that period, and for ten additional centuries, acted to return that potential energy to the earth. The referenced potential energy is that encountered in the construction and subsequent deterioration of stone walls. The Native American population is that of the Ancestral Puebloans occupying Chaco Canyon (36.06° N, 107.97° W) in what is now Chaco Culture National Historical Park (CCNHP), a U. S. National Park administered by the U. S. National Park Service (USNPS). The Park is also designated a World Heritage Site because of its prominence as a major cultural center of the Ancestral Puebloans in Southwestern North America.

The Ancestral Puebloans built massive sandstone buildings in Chaco Canyon, some of which may have been more than three stories high and contained more than 500 rooms. Those buildings, constructed between about 850 Current Era (CE) to the mid- 1100s CE, represent prodigious efforts requiring generations of artisans who prepared and incorporated hundreds of thousands of stones and thousands of tree parts into the structures. The present study explores why Pueblo Bonito, one of those massive buildings, did not survive the centuries by assessing structural conditions obvious on some of the earliest photographs of that Great House. I evaluated those structural conditions from the perspectives of the architect, the civil and mechanical engineer, the construction supervisor, the ecologist, and the archaeologist.

The hypothesis in this study is that "materials and technologies available to Ancestral Puebloan construction engineers favored the production of vulnerable walls and leaky roofs which contributed to roof failures and wall collapses at Pueblo Bonito". The hypothesis cannot be proven but the examination of some of the earliest photographs taken at Pueblo Bonito suggest several causes that may have contributed to the deterioration of the Pueblo Bonito Great House.

## Background

This section emphasizes the importance of Chaco Canyon as a World Heritage Site and also notates construction problems encountered by early Ancestral Puebloan engineers. There was a human presence in Chaco Canyon perhaps at least 5000 years ago (an organic sample from Atlatl Cave may carbon-date to 2900BC [1: 219]). Chaco Canyon structures, at least for some time, were central to Ancestral Puebloan culture in the San Juan Basin, even though other occupation sites occurred at high water table locations that very occasionally occur throughout the San Juan Basin [2]. Chaco Canyon, a high desert canyon apparently lacking sustaining surface water and presently considered a stressful habitat, may also have been near a high water table source. One hypothesis is that geological formations favored the development of a Hypocrene springs system just south of the South Canyon walls which provided a ground water ecosystem that was sustaining until eventual over-utilization effectively depleted wood and food growth resources [3]. Presumably, the Canyon vicinity during the times of early occupation provided agricultural production capabilities that now are absent. The present analysis strives to determine why stacked core masonry Great Houses, specifically in Chaco Canyon but perhaps representative of other Great Houses within the San Juan Basin, did not survive the centuries since construction. One structure, the ruins of Pueblo Bonito, has been selected as an example data source to assess probable causes of the deterioration of a Great House produced during the late Ancestral Puebloan habitation period.

The engineer and architect would say that, in addition to construction limitations, there were three types of natural stresses that might impact construction in the greater Chaco Canyon area- seismic, gravity, and wind. Seismic impacts are not considered herein as a significant cause of the deterioration of Pueblo Bonito because those events were distant in time and space.

There are examples of construction in antiquity where very large stones were cut with precision and interlocking pieces of large stones were set in place without mortar to form substantial walls or other structures. The cut precision was sufficient that the heavy weight of a top stone was transferred to an underlying stone through a large shared surface area so the "heaviness" was directed to the ground in an efficient manner that did not compromise wall strength. The effort in modern masonry is often to make the weight of stones on a top course pressure down on the next lower course, firmly pressing that course in place, and to collectively and efficiently push the weight of all stones to the ground.

Much masonry in antiquity and current architecture has used a lime-based mortar produced from a high temperature burning of limestone (CaCO_3_) which evaporates CO_2_, producing calcium oxide (CaO) which, when mixed with water, produces a plastic paste (Ca(OH)_2_) that adheres to sand and rock [4]. That paste acts as a bedding material which, after thorough mixing with the sand carrier and being applied to a wall, absorbs CO_2_ from the air through a carbonation process, binding the sand and rocks together into a limestone matrix. This type of mortar is especially useful because it allows stresses in a mortared stone wall caused by expansion, contraction, moisture migration, or settlement to be relieved by the mortar and not by the rock masonry [4]. It efficiently conveys weight from a top stone to an underlying stone by essentially "joining" the two stones.

The Ancestral Puebloans, though gifted in the use of rudimentary tools, were not able to cut large stones with great precision and did not have the ability to make lime mortar for binding stones into strong structures because the available building material was sandstone, not limestone. Very early habitation in Chaco Canyon emphasized the pit house and rock slab walls covered with clay/mud mortar. Even at this early stage, the artisans were likely to have been aware of the deficiencies of using clay/mud mortar on open walls where it would have dispersed in heavy rains. The structural deficiency of the available clay/mud mortar mix would have continued to be problematic for the Ancestral Puebloans throughout their residency in Chaco Canyon. This is a critical observation because mortar was the important connection for distributing gravity loads to great house foundations. The clay/mud mortar was likely the weakest part of the Great House wall, perhaps having only the strength and elasticity of an unfired clay pot.

Getting the weight of roofs, interior walls, floors, exterior walls, etc., to the ground without distorting and weakening walls and reducing wall strength is one of the great challenges in construction. Load paths describe how that weight is transferred to the foundation and load paths are considered in modern architecture [5] to help builders determine adequate anchors to attach roofs to exterior walls, walls to foundations, and to anchor or connect the foundation to the ground. Connections were an especially underdeveloped feature of Pueblo Bonito, often consisting only of the clay/mud mortar between courses of stone that bound walls together, to the roof, and to the foundation.

The forces and load paths that occur in the construction of a modern building using commercial cement blocks include a foundation of reenforced concrete stabilized into the soil, a cement slab poured around a rebar framework that has been secured into the foundation and laser-leveled to provide a flat, even surface for placing the cement blocks, which are then joined together with a lime-based mortar. The vertical walls may be aligned by laser or plumb lines and a roof framework of joists and rafters from dressed wood or metal bolted to a wall plate on a tie beam which, in turn, has been bolted to the tops of the cement block walls. The total weight of the roof, sheathed and surfaced from modern components, is relatively light and is transmitted downward through external walls or internal columns to the stabilized concrete foundation. In addition, 21^st^ century architects and builders consider a building as a six-sided box and their construction efforts use a wide variety of flashings, membranes, films, drains, other components, and management actions to keep unwanted water from each of those six sides [6]. Special attention is directed to waterproofing the wall-roof, and wall-foundation connections and safeguarding the foundation from water damage and soil subsidence. The modern builder benefits from pre-fabricated or lightweight construction items, a wide variety of hand and power tools, and directional manuals and designs. The Ancestral Puebloan engineer at Chaco Canyon encountered the same gravity, wind, and water problems facing the modern builder, but had to depend on the availability of heavy and bulky building materials, the need to shape stone and wood without ferrous tools, the need to move and manipulate building material without animal or mechanical power, and the complications of a deficient mortar.

## Methods

The architecture of the Pueblo Bonito great house in Chaco Canyon is inferred from archival photographs, the earliest of which date from the winter of 1887–8. The address of the online USNPS photographic data set is www.chacoarchive.org, and selected photographs from that data set are evaluated herein to help interpret how construction occurred at Pueblo Bonito and causes of deterioration. Pueblo Bonito was selected for this discussion because there are thousands of archival entries (about 4150) for that particular Great House. However, many of the conclusions of this study are presumed relevant to other Great Houses in the Chaco Canyon and the interior San Juan Basin. I selected 137 photographs from the data set because they seemed to offer the best examples of construction problems I deemed important for my analysis. They were selected from photos taken by Victor Mindeleff of the Bureau of American Ethnology of the Smithsonian Institution during the winter of 1887–8, photos taken by the Hyde Exploring Expedition (HEE) in 1896–1899, and photos taken by associates of the National Geographic Society beginning in 1921. The emphasis was to examine historical scenes before significant reconstruction and stabilization efforts were initiated by the USNPS, believing that the earliest photographs might better indicate causes of deterioration. The photographs are not reproduced herein but an accessible linkage to the photos is provided and identified in S1 Appendix. Some scenes need little explanation, e. g., an early overview of the Pueblo Bonito ruins (PB- 1), where it is indicated, as described in S1 Appendix, that it is the first cited photo in this study of Pueblo Bonito. Other images are information rich and are examined in detail, e.g., PB- 2 which shows missing core protection masonry around a window, blowout areas with missing core protection stones in several areas on the wall face, uneven sized stones within courses, wall settling, and stress cracks along vertical joint lines across the wall face indicating a dispersed load path and a weakened wall. The information about the individual photographs identified in S1 Appendix, within the www.chacoarchive.org data set, include: (1) image gallery; (2) site identified in the image gallery (in this case the Great House named Pueblo Bonito (PB)); (3) page number in the image gallery where the particular photograph appears and its position on that page; and (4) credit for the particular photograph and its archival identification.

This is a very focused study. It is an assessment based on photographs of Pueblo Bonito taken over about a 38 year period from 1887 to about 1925. The photographs in the database are a compilation from several collections, are sometimes duplicative, and reflect differences in quality of equipment, photographer skills, and differing areas of interest over the photographic study period. A few published photographs are also referenced. Pueblo Bonito suffered from some human activities after the departure of Ancestral Puebloans from the Canyon: vandalism (see caption PB-30), some undisciplined early archaeology, and some harvest of wood from within the great house [1: chapter 2]. The impacts of those activities on the present analysis are unknown.

This analysis of the structure of Pueblo Bonito: (a) describes the relationships between stone walls, mud mortar, water, rock faces, wood beams, wind, and biological agents; (b) notes the technical limitations encountered by the Ancestral Puebloan engineers; and (c) suggests that some past construction problems at Pueblo Bonito can be explained by modern construction experiences. An example of the logic in my analysis is: (a) there are photos that show patches of walls at Pueblo Bonito where stones are missing; (b) modern bricklaying masons provide for drainage in brick walls so water within those walls will not pond, freeze, and pop off bricks; (c) stone walls at Pueblo Bonito would accumulate water because of the porosity of the wall and its stones; and (d) presumably, freezing of that wall water would cause the observed displacement of stones from Pueblo Bonito walls. It is a logical assessment of a past action, based on modern experiences. The study is not a critique or history of the Ancestral Puebloans of Chaco Canyon.

## Results

### Mortar and gravity forces

Pueblo Bonito was a complex building consisting of many sandstone walls. Building a stone wall amasses weight and gravitational potential energy (gpe). A key to maintaining a wall is the maintenance of gpe, i. e., keeping the stones stationary within the constructed wall. Their weight needs to be conveyed to the ground in the same manner, over time, along a favorable load path. If that does not happen, the wall may eventually fail. Maintaining the favorable load path is not an easy accomplishment when working with stones of different sizes and masses. Deterioration of a stone wall may convert gpe to kinetic energy, a large change if deterioration leads to the fall of stones and the collapse of the wall, lesser changes if deterioration only involves shifting among rocks because some gpe is retained by those rocks remaining in the wall. The challenges for the Ancestral Puebloans who were building the stone walls of Pueblo Bonito were that the addition of each stone added potential energy to structural walls, while natural events like gravity forces, weather events (wind loads and precipitation), and biological agents were acting to destroy walls and return the gpe to the earth.

A brief history of Chaco Canyon exploration [1] summarizes James Simpson’s 1849 impressions of Great House ruins as " jumbled jagged walls projecting above mounds of fallen debris and windblown earth" and includes William Jackson’s 1877 depiction of Pueblo Bonito indicating that the West wall was quite ruinous and almost indistinguishable while the North wall was quite perfect and from three to four stories high [1]. Figs 20 and 21 in that book, Figs 4.3, 5.1 and 7.1 in [7], and image PB-1 in the photo gallery indicate the significant deterioration of Pueblo Bonito that had occurred before USNPS restoration began in the early decades of the twentieth century. Assumed causes of that deterioration are described below.

#### Foundation

The weight of the walls, ceilings, and roofs of Pueblo Bonito rooms was transferred to the ground through the foundation, which often was a low wall of rocks and mud mortar, partially buried in the alluvium of the canyon floor (PB- 3). Foundation walls would be top-leveled to present a flat surface for layering courses of wall rocks (PB- 4). Construction of some walls in Pueblo Bonito may have been on top of subsurface walls that were remnants from prior developments which were buried in the alluvium and dated to earlier centuries (PB- 5; PB- 6; PB- 7). Those earlier wall remnants may have provided a substantial anchor to the ground. Water flow or seepage through alluvium and porous sandstones could undermine and cause settling of foundations, leading to the breakage and collapse of wall segments (PB- 8; PB- 9), and be the cause of efflorescence at wall bases which might accompany stone and mortar erosion and deterioration [8]. An extensive foundation complex extending east from the northeastern portion of Pueblo Bonito (PB- 10; PB- 11) was never built upon, perhaps reflecting a growing concern by Ancestral Puebloan engineers of potential rock fall from the nearby northern cliff face, as happened in 1941, causing extensive damage to the northeastern portion of Pueblo Bonito (PB- 12).

#### The construction unit

Changes in wall construction over time include a trend to shape individual stones to attain greater symmetry in wall components, to better organize how stones were placed within the wall to increase wall strength, and to enhance the protection of the mud mortar holding the stones in place. Those changes are the subjects of the next few paragraphs.

The basic wall building units at Chaco were mud (a mixture of sand, silt, clay and water) and sandstone. Wooden beams and some other organic items were also incorporated into constructions as discussed below. Subsurface construction often featured a mudded wall with stones pushed into the mud mortar. Rock chips embedded in copious mud sometimes comprised the structure of foundation walls (PB- 3; PB- 17). A variation of that kind of masonry also occurred in subsurface walls. A best example (PB- 13) clearly shows chips incorporated into the mud to comprise such a wall. There are other examples (PB-14; PB- 16, PB- 18), and especially (PB- 15) which seems to be a mud wall with embedded stones, that has been plastered or mudded over. These examples survived because their subsurface position lessened their exposure to moisture.

**Fig 1:**
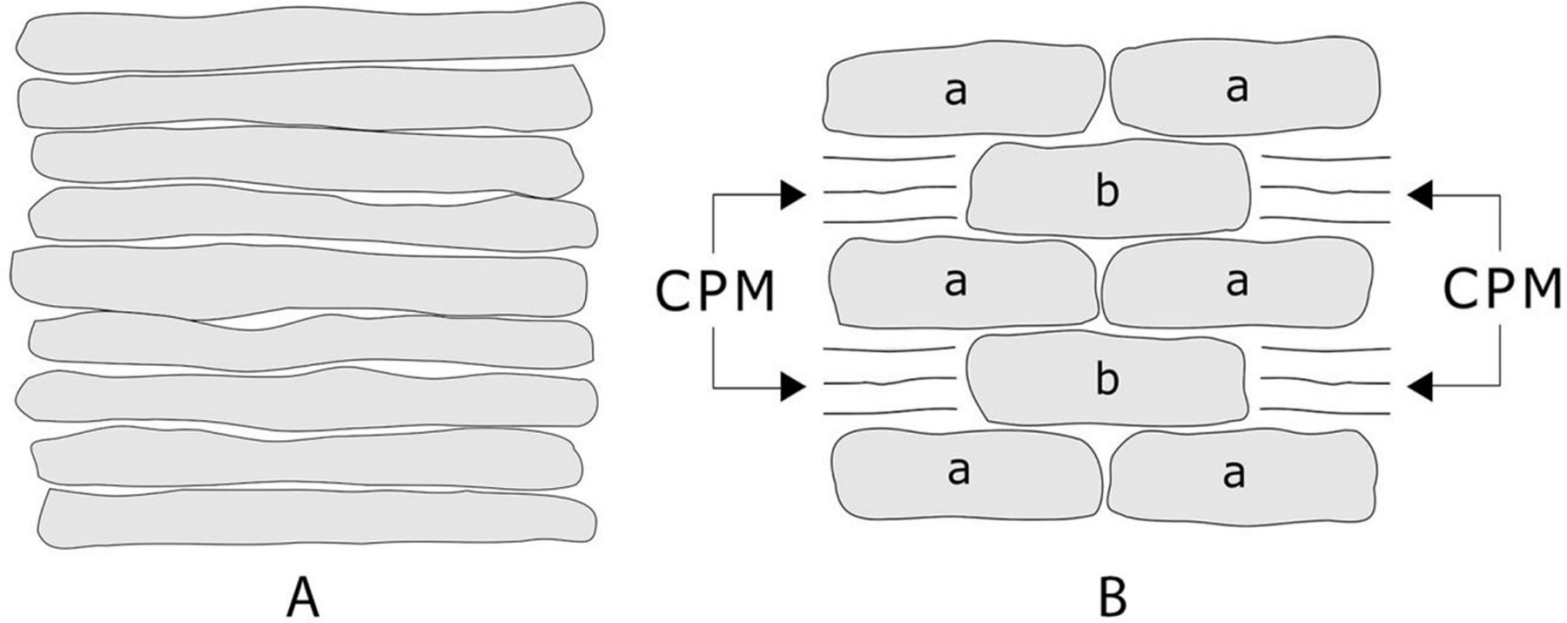
Examples are stacked plate masonry (A) and stacked core masonry (B). Stones marked (a) in example (B) would correspond to "headers" in contemporary brick masonry. Stones marked (b) in example (B) would correspond to "stretchers" in modern brick masonry and core stones in Ancestral Puebloan masonry. CPM (core protection masonry) in Ancestral Puebloan masonry consists of slabs wedged between courses of stones (a) to provide levelness and to protect the mud mortar.

Above-ground surface walls, exposed to the elements, used mud to bed the stones, with stones overlaying other stones, becoming the important wall volume. The challenge in these above-ground surface walls was protecting the mud mortar from moisture. Early stacked plate masonry surface walls (Fig 1A, is a representation of stacked plate masonry) were one stone wide (PB-19), and photographs (PB- 20; PB- 21) show layers of different sized rocks dipping in disorderly courses, providing uneven between-rock contact and diffused load paths. Some stacked wall plate segments standing in 1887 had fallen by the time of the Hyde expedition of 1896–9, indicating the continual process of wall deterioration. Presumably, the individual stones had been embedded in mud mortar, but the mud substrate had weathered away over time. Thus the problem: the mud-binding unit was unable to maintain wall integrity because the mud was susceptible to dispersion by rain, flooding and wind.

#### The core

To build above-ground height, the Ancestral Puebloan engineers needed to add strength to the walls beyond that available in a single stone-wide stacked plate masonry wall. That additional wall strength was necessary to attain a stability that would retain the accumulating potential energy. Simply laying two stones side by side and mudding them together (PB- 22) would only be a nominal improvement over single stone-wide stacked plate masonry, but tucking the ends of the plates under or over one another, or better yet, laying other stones over the touching plate ends so the plates could not shift, and thus would be confined, would provide strength. The product was stacked core masonry. Examples are (PB- 27; PB- 28) as well as representation B in Fig 1. The photographs identified as examples of stacked core masonry, cited below, are imperfect because such illustrations require wall cross-sections and such cross-sections exist only because of wall trauma. Stacked core masonry construction usually used larger stones with many smaller stones wedged in to provide stability and levelness (PB- 25). An additional technique was utilized in the construction of the great north wall which was a meter wide at its base. The core contained rows of both flat plates, large core stones, abundant mud, and rock debris (PB- 29; PB- 30; PB- 31).

#### Protecting the core

There are similarities between the modern brick wall and the stacked core masonries of Pueblo Bonito. Bricks comprising a course called "headers" are analogous to stones identified as (a) in Fig 1B, and bricks called "stretchers" are analogous to stones identified as (b) in Fig 1B. The 21^st^ century mason fills the height gap between courses of "headers" with partial bricks and lime-based mortar. The Ancestral Puebloan mason, dealt with asymmetrical "headers" and "stretchers", a mud mortar, and resorted to filling the height gap between courses with mud and rock fragments (the CPM in Fig 1B) to provide levelness before the next course of stones (a) was added. That CPM filling is called core protection masonry herein. If the wall grew wide by adding additional "headers" and "stretchers" internally, then there were internal spaces to also be filled with a jumble of rocks, rock fragments, mud, and debris (PB- 24; PB- 26). Such rubble filling seems common in some Pueblo Bonito walls. A core consisting of rocks with mud would be vulnerable if wetted, with the loss of the binding-mud resulting in the shifting of rocks with uneven surfaces, reducing the contact between rock surfaces and causing a dispersed load path. A good strategy to avoid this problem was to protect the core with CPM to produce a stable load path.

Core protection masonry (CPM) involved positioning external stones to minimize the exposure of the mud mortar to moisture. It provided a rock surface covering that made stone walls less porous and more stable. The CPM used has frequently been referred to as a veneer, but it was not technically a veneer because that is a cladding supported by a wall and the CPM was stonework in support of the wall. The modern mason would use bricks or brick parts and lime-based mortar to fill and prepare for the next course. The Ancestral Puebloan mason eventually settled on abraded surface stones, to fill and prepare for the next course, with interior end surfaces of some of the CPM stones wedged beneath core rubble stones (PB- 27; PB- 28 –see wall cross-section; PB- 29). The core with its core protection masonry would comprise many walls at Pueblo Bonito.

#### Evolving technologies

The intended evolution of CPM was to minimize the area of exposed and vulnerable wall surface mud by using more symmetrical stones, so in a unit of space there would be fewer stones and greater areas of contact between those stones in a vertical plane. At least some of that general methodology can be seen in different CPM examples ("Types of masonry") at Pueblo Bonito. A "Type 1" masonry sample (PB- 21) consisted of one stone wide courses with the presumed mud mortar weathered out. It is considered representative of early construction. "Type 2" (PB- 32; PB- 33) and "Type 3" (PB- 34) masonries only differed in detail. Both contain courses of large irregular sized rocks with narrow slabs wedged in to level courses ("Type 2"), and somewhat thicker slabs to fill such spaces ("Type 3"). Each employed a complicated geometry to help distribute heavy wall weight but probably did it ineffectively. Those configurations did not provide optimum contact between the stones on different courses. Should the mud mortar be lost, stones would shift, and load paths would become dispersed. The CPM corresponding to "Type 4" masonry (PB- 35) featured abraded plates arranged in symmetrical courses, with increased surface area contact between vertical rock courses, suggesting that wall weight from core protection stones might be directed downwards more efficiently. It may be, that through trial and error, it was discovered that this CPM form or similar masonry types provided better protection to wall and core mortars and greater levelness to the wall. Both form and function benefitted from such masonry. The greater consistency and symmetry in this CPM resulted in very aesthetically pleasing walls (PB- 35) and enhanced load paths and improved stability to wall courses. Other authors have suggested that the "Types" of CPM reflect different construction sequences at Pueblo Bonito or the influence of different societal groups into Chaco Canyon [9: p. 200]. There are many instances where different types of CPM appear on the same wall at Pueblo Bonito, suggesting that building, repair, and reconstruction of that wall occurred over the time of occupancy.

#### The environment

The Ancestral Puebloan builder needed to keep stones stationary within the constructed wall, while working with stones of dissimilar sizes and shapes held in place by being wedged beneath other irregular stones, while using a mortar that was vulnerable to water saturation and persistent dampness which caused the breakdown of the clay matrix, leading to a loss of structural integrity [10]. If the mortar, intended to provide a stable substrate for stones, became friable after becoming wet, stones could shift positions so that a stone with an uneven surface could wedge against other stones with uneven surfaces, altering load paths. Courses of stones could become uneven if CPM stones were of uneven size (PB- 36; PB- 37), core stones or CPM stones shifted because of mud loss or viga loss (PB- 38), or foundations shifted (PB- 8; PB- 39), with contorted gravity load paths often appearing as wall cracks, both on external walls (PB- 2; PB- 28; PB- 40; PB- 41; PB- 42) and interior walls (PB- 43).

#### Moisture

Water leaks into the wall could impact walls in two ways. Pooling within a wall, or water pressures against or through a wall, could loosen and wash out rocks (PB- 44; PB- 45). Additionally, water that penetrated a wall could pond, freeze, expand, and blow out segments of walls (PB- 2; PB- 24; PB- 46; PB- 47; PB- 48; PB- 49; PB- 50; PB- 51), sometimes causing segments of wall to shift (PB- 52; PB- 53; PB- 54) or architectural features like doorways to lose symmetry (PB- 55; PB- 56; PB- 57). The appearance of wall blowout areas further indicates that CPM was structural because supported stones over the blowout area would have collapsed had that portion of the wall been veneer cladding. Some wall segments seem to have just disintegrated because of catastrophic failures (PB- 23; PB- 39; PB- 58; PB- 59; PB- 60).

#### Construction deficiencies

There are examples of construction at Pueblo Bonito where two walls just abutted at a corner rather than being integrated or dove-tailed together (PB- 61; PB- 62; PB- 63; PB- 64), where the spacing between wall connections was filled with right-sized stones (PB- 65), or where different CPM types just abutted (PB- 63), resulting in irregular courses and wall cracks (PB- 66; PB- 67). In some cases an intramural wooden beam was used to try to join two walls (PB- 68), providing a weak structural connection between wall segments. Such construction deficiencies might be expected since Pueblo Bonito was built across centuries. However, they still represent areas with weakened load paths and strongly suggest that the additional room and wall constructions were not part of any original master plan. For example, rendered plans of Pueblo Bonito [7: Figs 5.5; 5.7] indicate a general lack of symmetry in the overall design and many odd-shaped rooms that likely would not have been planned space. There are, however, several instances where segments of standing walls seem to defy gravity (PB- 69; PB- 70), attesting to the construction of some strong load paths.

#### Special wall construction

The modern building inspector likely would not approve a free-standing brick wall, on a cement foundation, that was greater than 3 or 4 m in height. But the great north wall at Pueblo Bonito was 13 or 14 m in height. Such a wall, built of irregular sized stones on an infirm foundation, with a mud mortar, was an engineering feat requiring ingenuity and imagination. The remnant great north wall of Pueblo Bonito may be the remains of a double wall with the inner southern wall much degraded. Images (PB- 71; PB- 72; PB- 73; PB- 74; PB- 75; PB- 76; PB- 77) clearly show part of a parallel wall with intermural connections. Archaeologists have noted [7: p. 12–13] that rooms nearest the north wall, i. e., furthest from the southern plaza, were wide E-W, but narrow N-S, and not connected frontally (southerly) to other Pueblo Bonito rooms. The double wall may be a construction technique with intermural walls tying the two walls together, essentially producing a series of cells whose presumed function was structural support. Holes were present near the top of the north wall where vigas (a roof-beam like structure) had collapsed and leveraged out rocks surrounding the sockets (a wall hole holding the viga) (PB- 78). That indicates that the two walls at one time were equally tall because the vigas horizontally spanned space. The double wall structure thus had a front (the northern wall) and back (the southern wall), presumably sides (the intermural walls), and the indication of a roof, suggested by spaces where roof vigas formerly existed. That configuration suggests the north wall may be a remnant of what essentially was a long and narrow building. That may have been the necessary construct to develop and support a wall 13 m or so in height. The base of the north wall was broad with a thick core, faced with CPM (PB- 31; PB- 79; PB- 80). Presumably that wall width was reduced as wall height increased (PB- 81). The southernmost of the two walls was connected to, but did not open into, other Pueblo Bonito rooms (PB- 72) which were directed towards the plaza. Upper story rooms bordering the southern wall construct may have been intended as supporting structures for the southern of the two great north walls. A repaired and stabilized portion of the double wall shows the narrow passageway between the two walls and the presence of the intermural walls (PB- 82; PB- 83). It is not known whether the double wall structure had a symbolic relationship with the neighboring tall canyon wall but a 4 or 5 story wall was not a necessary feature of a 2 or 3 story building. The north wall was periodically rebuilt and repaired during the time of Ancestral Puebloan occupancy judging from the different forms of CPM present (PB- 84).

#### Wood

Wood was used extensively at Pueblo Bonito because it was manipulable and could span space. Wood survived differently through time depending on whether it encountered an aerobic and moist environment or occurred under dry-anaerobic conditions. Wetted roof vigas and wood rot are described below as major contributors to top-side roof and eventual wall collapses. Remnants of wetted wood appear as deteriorated upright stakes in jacal construction where mud was plastered between the stakes (PB- 85; PB- 86). Sometimes wooden lintels over doors or windows warped or broke, presumably because they were wetted and rotted, causing displacement of stones in courses (PB- 87; PB- 88; PB- 89), thereby disrupting load paths. There is at least one instance of a wall collapse where that wall was built on a beam that eventually decayed (PB- 90), presumably because it had been wetted. A second wall disintegration photo may also involve a decayed beam (PB- 23).

Intramural beams in non-wetted conditions provided horizontal stability within wall courses. Sometimes their remnants were evident, protruding into space after a wall portion had fallen (PB- 91). Such beams were used to separate rooms (PB- 92), provide vertical stability (PB- 93), support stone walls (PB- 94; PB- 95; PB- 96; PB- 97), and sometimes they just appear in excavated rooms serving unknown functions (PB- 98; PB- 99). Intramural wooden beams were sometimes used to strengthen the connection between wall segments (PB- 100). Wood was also used extensively in flooring (PB-101; PB- 102), in the complex overlaying of many timbers to form the roofs of kivas or round rooms (PB- 103; PB- 104; PB- 105) where numerous timbers apparently survived because they encountered conditions unfavorable for wood rot, and as dressed wood in post and beam framing (PB- 106), especially around windows and doors (PB- 107). The importance of wood is further emphasized by the many photographs of broken roofs and fallen ceilings (PB- 108; PB- 109; PB-110; PB- 111). Images also indicate a variety of ceiling constructions, e. g., pine vigas (roof, joist-like structure) covered with latillas (sheathing-like structure) (PB- 113), including sapling latillas (PB-116); willow twigs (PB- 112); willow saplings covered with round reeds stitched together with fiber (PB- 114); peeled willow saplings covered with flat reed leaves over pine vigas (PB- 115); and other miscellaneous ceiling components (PB- 117; PB- 118; PB- 119).

#### Sequence

The word implies time and time implies change. Construction at Pueblo Bonito may have been in spurts [7], or more or less continuous, but deterioration was continuous. Some causes of deterioration were time-independent; e. g., water entry into walls could occur any time after construction, impacting wall muds and distorting load paths, and subsequent freeze cycles with accumulated wall water could cause wall blowouts. Time-dependent deterioration might be viga wood rot, believed likely responsible or contributory to roof and wall collapses described below and which may have required decades of incubation. Some wall damage was irrepairable (PB- 2; PB- 48; PB- 51), but other walls could be repaired, and clearly were, perhaps several times. Some wall sections were repaired with CPM different from the original masonry (PB- 121; PB- 122), before the wall segment finally became irrepairable (PB- 120). Presumably, if the walls and roofs of a room could not be repaired, that space became an abandoned portion of Pueblo Bonito.

Archaeologists have described building sequences at Pueblo Bonito on the basis of masonry types. Ground plans of early (PB-123) and late (PB- 124) constructions at Pueblo Bonito are presumed to reflect constructions across time. The details of early descriptions have been somewhat modified in later text documents [7: Fig 5.7] which propose construction sequences in greater detail. Areas of the Pueblo Bonito complex exhibit different types of masonries, suggesting different construction eras or engineering capabilities (PB- 68; PB- 100; PB- 125; PB- 126; PB- 127). Whether some subsurface round rooms were efforts to produce stronger wall structure is unknown but, in general, those subsurface walls seemed to have fared better than walls in above ground structures (PB- 128; PB- 129; PB- 130), perhaps because the accumulating over-burden of aeolian debris shielded them from water action.

### The wind

A second type of force or pressure exerted upon a building, in addition to gravity, is that produced by wind. During spring, the average wind speed at CCNHP is 16 kph (10 mph) with velocities exceeding 40 kph (25 mph) about 1% of the time. Windstorms lasting one to two days with velocities of 60–80 kph (40–50 mph) sometimes occur, as do dust storms during springtime [8]. Studies have described how wind impacts buildings and the windward wall, where wind strikes [11]. If the wall is non-porous, e.g., the cement block wall described above, the winds are deflected upwards, downwards, and sideways. The laminar flow over a flat roof may produce an uplifting suction effect on the roof. If the building has a very complex envelope, then the winds over the building may become turbulent. If the windward wall is porous, meaning that wind passes into the building as well as being deflected at the wall, then the wind can produce both positive and negative pressure gradients within the building [11]. Impacts from changes in air pressures within above-ground rooms of Pueblo Bonito during wind events would be insidious rather than blatant. If walls were very porous, then winds would blow adobe mud or clay coverings from some interior walls (PB- 87; PB- 131; PB- 132), probably blanketing certain internal surfaces in thick dust.

Wind loading, and air turbulence above a roof, would be important to the maintenance of the roofs of Pueblo Bonito and to the integrity of roofless walls. The heavy roof exerted a downward gravity force on the vigas (a structure corresponding to roof joists on a modern flat roof) within their sockets, and those same vigas and the latillas (a structure along with splints that would correspond to the sheathing on a modern roof) were periodically experiencing an upward force if the building or room became pressurized. Changing air pressures likely would not provide enough force to move the heavy vigas within their sockets, but pressure changes and air turbulence above the roof could contribute to the cracking of external roof-wall surface connections which were of adobe and mud. That would mean that the important connection between the wall and roof could be severely compromised and weak. Cracks at roof corners or along the roof perimeter would allow the entry of water.

### Roofs

Neo-Pueblo construction, as frequently seen in Southwestern North American cities, features flat roofs or near-flat roofs with only minor elevations to facilitate water runoff. When well constructed, such low pitch or flat roofs can be expected to prevent leakage for perhaps 20–30 years. The roof, even in the 21^st^ century, is often a problematic part of a structure, but the intent is to produce a structurally light cover over a substantial building frame.

The great challenge for the Ancestral Puebloan architect would be keeping water from precipitation out of the 500 rooms of Pueblo Bonito. A good solution would have been to build a pitch to above ground roofs that enhanced drainage, but that would not have been easy.

The 21^st^ century architect studying Pueblo Bonito would be troubled by the apparent contiguity of top-side rooms with individual roofs. Individual rooms, at least as presently considered, appear as cells in Pueblo Bonito floor plans [7: Fig 5.5], (PB-124). Each cell-like, top-side room presumably had their own flat roof without obvious drainage capabilities and, presumably, common walls with sockets holding vigas serving contiguous rooms. That forced a no pitch, flat roof design in the form of a large number of individual roofs of differing ages because Bonito’s rooms and roofs were built over different decades or centuries. Whereas the neo-Pueblo flat roof has internal or external drains built to expedite water removal, the heavy flat roofs of Pueblo Bonito seem to have relied on evaporation in the arid environment of the canyon to remove water. Until roofs dried, they acted as clay-covered evaporation pans with leaky edges, the edge cracks being the product of varying wind pressures, and surface cracks where water dissolved surface muds or freeze-cracked the surface. The images (PB- 72; PB- 74) which show many broken walls without roofs may reflect common roof failures. However, it must be pointed out that it is not known if some of the missing roof beams were due to the human harvest of wood from Pueblo Bonito after the exodus of the Ancestral Puebloans.

One surviving roof (PB- 133) illustrates the massiveness of the Ancestral Puebloan wood-earthen roof. Roof descriptions have also been derived from roof remnants that fell onto floors (PB-108; PB- 109). At least one roof may have become a museum acquisition (see caption, PB- 134). An Ancestral Puebloan heavy, no pitch, flat, earthen roof frequently rested on vigas that were generally ponderosa pine (*Pinus ponderosa*) logs 20–25 cm in diameter [12], each perhaps weighing over 50 kg. Vigas were usually placed within wall sockets with the supporting stone wall built up around them. The vigas had the important duo role of supporting the roof and possibly helping to keep the walls from collapsing inwards. Latillas were smaller rounds of pine or other woods (5–10 cm in diameter) that were placed perpendicularly across the vigas. Roof vigas and latillas were covered with splints frequently split or stripped from junipers (*Juniperus* sp.) [12]. The top roof covering of layers of adobe/mud, sometimes combined with bark, rocks, and earth, would correspond to the tile, shingles, or membrane of a modern roof. Such an Ancestral Puebloan roof, 18 m^2^ in area, might weigh 6000 kg [13], (about 68 lbs/ ft ^2^), while some modern flat roof coverings weigh less than 5 lb/ ft ^2^. A photograph exists of a portion of a heavy and broken Ancestral Puebloan no pitch, flat, earthen roof that has collapsed into a second story room [14: p. 82], although that photo is not from Pueblo Bonito. The supportive strength of the stone walls would have limited the size of a roofed room because of the inability of the walls to provide the necessary stability and support to the great roof weights at Pueblo Bonito.

#### The leaking roof

The top-story roofed area of Pueblo Bonito presented a very complex envelope in time and space, especially if roofed rooms were contiguous in placement. As room construction occurred over decades and centuries, protective and contiguous roofs would vary between new and aged. Eventually, most or virtually all above surface roofs failed and there likely was a common element to that failure. Water, such a necessity to life, can be death to a leaky roof that is supported by untreated wood. The combination of wetted vigas from water leaking through cracks in the roofing materials and around roof corners and perimeters, moderated temperatures under an insulating earthen cover, oxygen, and darkness or dim light would have allowed wood rot fungi, whose reproductive spores were in the air or in the earthen roof cover, to reach the wetted wooden vigas, reproduce, thrive, and digest the cellulose, lignin and other cell wall components, thus materially weakening the viga’s structure [15]. The critical moisture level in the viga needed to be about 27% of its dry weight for wood rotting organisms to digest and effectively impact the molecular structure of the wood fibers. Once started, deterioration might continue as the breakdown of the viga’s carbohydrates in the fungi’s respiration process produced water and carbon dioxide [15], thus maintaining wetted conditions favorable for rot, which would cause a weakening or a loss of toughness in the viga. That loss in toughness (weight bearing capability), because of breakage of chemical bonds in wood fibers, was disproportionally greater than was the viga’s weight loss through decomposition [16], meaning a viga could look strong but act weak. Woody debris from fallen roofs or ceilings, e.g., (PB-108), neither looks burned nor discolored by rot. Rather the debris looks fatigued and shattered, with a major beam appearing almost flaccid, as might happen with an over-loaded beam that had lost tensile strength.

Intramural poles (PB- 93; PB- 100) were often covered with mud and rocks within rock walls which provided them with an anaerobic and dry covering unfavorable to rot. As a result many of those beams could survive, perhaps to even be used in subsequent Great House developments, while wetted support vigas partially exposed to air might rot. Some unknown number of the numerous vigas were harvested for construction or firewood after the exodus of the Ancestral Puebloans from the Canyon, complicating any further assessment.

The function of a roof can be thought of in terms of a water balance with the ideal balance for many roofs being precipitation = runoff. Flat earthen roofs would have been high maintenance structural elements because they would have developed cracks and lost soil to winds, while water would have puddled, ponded, channeled, and sometimes leaked into the structure. The water balance on such roofs would have been precipitation = evaporation + leakage. Maintenance would have required additional clays, muds, and other roof components to be periodically added to the roof to cover cracks at the wall-roof boundary and elsewhere on the surface to prevent further leakage. As the roof became heavier because of the addition of soils to control water leaks, the supporting vigas would have become less tough and less capable of supporting the weight of the heavy roof because of the loss of tensile strength as a result of wood rot. The result would be the continued deterioration of the viga until it no longer could support the heavy roof, which would then collapse (PB- 108). Sometimes the crashing movement of heavy but weak vigas during roof collapse leveraged out stones around viga sockets, producing gaping wall holes (PB- 78). The walls, after roof collapse, were vertical piles of stone rather than a part of a structure. In that condition, they provided less resistance to wind and rain and became more susceptible to continuing collapse. Modeling [17] has suggested another potential structural problem with the Ancestral Puebloan heavy, no pitch, flat, earthen roof. The stress distribution from a load, like a heavy roof supported by a heavy viga resting in wall sockets, with no socket support from intramural beams, would be transmitted downward in a diffused manner, producing a dispersed load path and masonry wall weakness that approached instability. Presumably, whether wall weakness was from physical stresses within stone walls caused by a diffused load path, weak wall-roof connections, storm damage to roofs and walls, or because of viga failures from wood rot, problems with the heavy, no pitch, flat, earthen roof likely contributed significantly to eventual stone wall collapses throughout Pueblo Bonito’s history. Some wall segments (PB- 21) photographed by Mindeleff during the winter of 1887–8 had already collapsed by the time of the Hyde Expedition in 1896, and other wall segments photographed by Mindeleff had collapsed before 20^th^ century restoration could occur (PB- 135).

The disintegration of a wet wooden viga under an earthen roof has some relationship to the rate of disintegration of a fallen tree on a forest floor. One forest model applicable to eastern U. S. forests has estimated that logs of many coniferous species on a forest floor lose 50% of their original biomass within an average of 18 years and are completely decomposed within 57–124 years [18]. The longevity of a wetted wooden roof viga in Pueblo Bonito might have been some small multiplier of those numbers, but the viga did not have to completely decompose, only lose significant toughness or weight- bearing capability, to become a structural problem. Ancestral Puebloans would have experienced roof and wall failures even if the wetted viga infested with wood rot fungi lasted as long as a century before collapsing.

## Discussion

An obvious question is whether any current conditions, acting over a similarly long time period, could have produced the roof loss and wall failures observed on the archival photographs. Also, whether something mimicking the present impacting conditions likely occurred during the time of the Ancestral Puebloans. The most obvious answer is that the basic cause of deterioration seems to have been the inability of Ancestral Puebloan engineers to control the impacts from the limited annual precipitation, presently measured at about 220 mm per year. The incongruity is that the precipitation that was likely insufficient to provide necessary agriculture within Chaco Canyon may have been sufficient to be causal in the deterioration of the Ancestral Puebloan massive stone structures. The deterioration effects were the results of many small events, such as the rotting of a viga or a shifting of wall stones, that constantly occurred over decades. The mud mortar was always inadequate unless it was protected in subsurface chambers or in sealed wall cores, in each instance out of contact with moisture. The mud mortar provided weak connections within courses of masonry and between that masonry, the roof, and the foundation.

Piling stones together was a good way to build a wall and the use of the mud mortar was necessary to bed the stones so that some measure of levelness and permanence could be attained in the wall. The problem was always to keep water away from the mud mortar so the bedding material was retained, stones remained in place, potential energy was conserved, and the wall retained stability. The Ancestral Puebloans were hindered by their reliance on the mud mortar.

A construction progression at Pueblo Bonito can be traced from a wall one stone wide to a core design where stones were laid from both the inside and outside of the wall with ends tucked under core stones to provide additional stability. The core became integral because it allowed walls to be built higher and functionally last longer than could occur or be accomplished with single stone-width stacked plate masonry. The core protection masonry (CPM) increased form by constraining the core from wandering and provided function by protecting the mud mortar within the core. The different CPM types ("Types of masonry") were likely efforts to find that design that most effectively protected the core mud mortar. It may have been a fortuitous effort- the designs that became most aesthetically pleasing also provided the greatest protection to the mortar. The wind, blowing from West to East down the Canyon may have contributed to the very extensive West wall failure. Otherwise, the wind, usually light to moderate in velocity, pecked away at exposed friable mortar, and both winds and rain increased the porosity of windward walls leading to the loss of interior wall veneers of adobe and plaster. Air pressure fluctuations within, and air turbulence above the building, only had to be sufficient to help promote flexing and cracking at the flat roof-wall connection and to aid moisture penetration through the flat roof-wall connections, across the roof surface, and through windward walls to contribute to building deterioration. Water passage from wall tops, between stones on masonry courses, and through the sandstone wall rocks could lead to water accumulations within walls causing wall washout events. Freeze cycles would lead to wall "blowouts" where external wall facings would be lost, causing increased vulnerability to wall cores. Such wall cores, exposed and washed by rains, would lose mud mortar, causing core rocks to shift positions, leading to dispersed load paths and further chances for wall failure and collapse.

The roof is frequently the most vulnerable portion of a building. The roofs of Pueblo Bonito were heavy and bulky, because suitable light weight materials were not available. The Ancestral Puebloans were not successful at constructing a lightweight, long-lasting, water-proof, roof. Vigas supporting the roof were built into wall sockets, and the vigas and over-laying latillas probably had the dual function of helping to provide shelter and acting as spreaders, keeping walls from collapsing inwards. Roofs by their structure could undergo some repair, e. g., mud filling of cracks across the roof surface and at roof-wall connections where cracks may have been caused by wind pressure changes. A serious design flaw may have been that roof-top rooms were built in a contiguous design so that a common wall may have been used to support vigas and roofs in contiguous rooms. That would mean that any common wall that was supporting vigas and roofs for two different rooms could not be easily replaced or substantially repaired. Presumably, the Pueblo Bonito wall had a dependency and a life time- the time required until wood rot to wetted vigas reduced viga tensile strength to levels incapable of supporting the heavy roof. Walls without a roof would be vulnerable to wind, rain, loss of mud mortar, and stone shifting, resulting in losses in potential energy, the production of diverse load paths, instability, and eventual collapse. The general sequence may have been rain, roof and wall leakage, viga rot, roof collapse, wall collapse, and perhaps, and then only rarely, significant wall and roof repairs.

The time-independent stresses that the Ancestral Puebloans frequently experienced might seem to be inconveniencies, but the water leakage into walls would wet core mud and eventually lead to disrupted load paths and possibly wall blowouts. Likewise, the leakage that wetted vigas and beams would in turn seem inconsequential events by themselves, but that leakage provided the environment for wood decay, which in a human lifetime or two, could be causal of roof and wall collapse. Together, the impact from the time-independent and time-dependent deteriorations would make building repairs necessary, but some repairs were impractical or impossible, presumably leading to the abandonment of severely damaged rooms. The history of Pueblo Bonito may have been that rooms with their individual roofs were constructed over a three-century period, and every construction cycle within those three centuries was followed a few decades later with a failed roof cycle with associated wall damage. It is a plausible scenario because the wood rot fungal agent was present, the wetted roof viga environment was favorable for wood fungal growth, and the timeline was suitable for the roof failure cycle to have been part of the history of the Pueblo Bonito Great House. The architectural cycle at Pueblo Bonito may have been room construction, roof and wall failure, room abandonment, followed by new room construction.

## Conclusion

The present day visitor to Chaco Canyon does not see what Simpson, Jackson, Mindeleff, and members of the Hyde Exploring Expedition observed in the 19^th^ Century. Significant stabilization and reconstruction work using modern masonry techniques was accomplished by the USNPS in the early 20^th^ century (PB-136) to produce a weather-proof building-scape, which has in turn generated designs thought to possibly have resembled old Pueblo Bonito. The Ancestral Puebloans in the early 1100s, however, likely never saw the magnificent Pueblo Bonito structure suggested from those 20^th^ Century designs that are imagined on modern pamphlets, brochures, or drawings (PB- 137), which condense into a single image, the presumed results of three centuries of construction. Such images of Pueblo Bonito are misrepresentations because they do not incorporate the continual structural destruction to roofs and walls that occurred over that same three century time period. Indeed, it is possible that some new constructions that periodically appeared at Pueblo Bonito were intended to replace failed building units that had become unusable, meaning that the habitable portion of the building could have constantly changed over the time of Ancestral Puebloan residency within Pueblo Bonito.

## Supporting information

S1 Appendix (DOCX)
