# Supplementary material for: The deterioration of the Pueblo Bonito Great House in the Chaco Culture National Historical Park, New Mexico, USA
Source: PLoS One. 2022 Apr 5;17(4):e0266099. doi: 10.1371/journal.pone.0266099 (PMC8982885; doi:10.1371/journal.pone.0266099)
Supplement: S1 Appendix — (DOCX) [file pone.0266099.s001.docx]

**S1 Appendix**

The location and identification of cited photographs in (**www.chacoarchive.org**) are (1) image gallery, (2) site identified in the image gallery (in this case the great house named Pueblo Bonito (PB))(3) page no. where the particular photograph appears and the position of the image on that page; and (4) credit for the particular photograph, and its archival identification. For example, an early photographic overview of the Pueblo Bonito ruins (PB- 1), occurs on p. 19; #1,- indicating it is the first image on page 19 of the PB image gallery site, where the originating photo is identified as an overview of Pueblo Bonito. It is identified as Maxwell Museum of Anthropology Collection, MAX 88_42_28.

**Important abbreviations**

CCNHP Chaco Culture National Historical Park

AMNH American Museum of Natural History

NAA National Anthropological Archives

MARI Middle American Research Institute, Tulane Univ.

HEE Hyde Exploring Expedition

NGS National Geographic Society

MAX Maxwell Museum of Anthropology, Univ. New Mexico

**The cited photographs**

PB-1. p. 19; #1 MAX Museum Collection, MAX 88_42_28

PB-2 p. 106; #20 CCNHP Museum Collection, Chaco Archive A0079800, HEE 404

PB-3 p. 202; #12 CCNHP Museum Collection, NGS negative #32000, slide 64

PB-4 p. 202; #10 CCNHP Museum Collection, NGS negative #31995, slide 63

PB-5 p. 1; #18 AMNH, AMNH accession #000923, HEE Granger 175

PB-6 p. 6; #20 AMNH, AMNH accession #000920, HEE 208

PB-7 p. 202; #14 CCNHP Museum Collection, NGS negative #38976, slide 71

PB-8 p. 83; #4 NAA, NAA judd_ngs_0883, NGS 22653-A

PB-9 p. 83; #12 NAA, NAA judd_ngs_0882, NGS 22688-A

PB-10 p. 202; #9 CCNHP Museum Collection, NGS negative #32356, slide 65

PB-11 p. 202; #8 CCNHP Museum Collection, NGS negative #28624, slide 60

PB-12 p. 179; #3 CCNHP Museum Collection, Acc. # 521, PB 78233, CHCU 52336

PB-13 p. 51; #11 NAA, NAA judd_ngs_0699, NGS 11893-A

PB-14 p. 31; #15 AMNH, AMNH 412012, HEE 266

PB-15 p. 35,; #1 AMNH, AMNH 412078, HEE 352

PB-16 p. 5; #15 AMNH, AMNH accession #000921, HEE 244

PB-17 p. 200; #14 CCNHP Museum Collection, NGS negative #28524, slide 23

PB-18 p. 121; #19 NAA, NAA judd_ngs_0804, NGS 28469

PB-19 p. 107; #8 NAA, NAA judd_ngs_0809, NGS 11851-A

PB-20 p. 9; #16 NAA, NAA judd_mindeleff_005, NAA 3015

PB-21 p. 13; #20 NAA, CRA accession #001284, NAA pl14_nm_173

PB-22 p. 72; #17 NAA, NAA judd_mindeleff_040, NAA 3301

PB-23 p. 72; #2 NAA, NAA judd_ngs_0797, NGS 11961-A

PB-24 p. 77; #18 NAA, NAA judd_hyde_317, HEE 652

PB-25 p. 71; #11 CCNHP Museum Collection, Chaco Archive A0079952, HEE 395

PB-26 p. 88; #7 NAA, NAA judd_ngs_0838, NGS 34493-A

PB-27 p. 83; #2 NAA, NAA judd_ngs_0906, NGS 50592-A

PB-28 p. 84; #14 NAA, NAA judd_ngs_0876 (wall cross-section), NGS 16270-A

PB-29 p. 82; #13 NAA, NAA judd_ngs_0723, NGS 11951A

PB-30 p. 82; #7 NAA, NAA judd_ngs_0729, NGS 23233A

B-31 p. 200; #6 CCNHP Museum Collection, NGS negative # 23233, slide 14

PB-32 p. 5; #2 NAA, NAA judd_ngs_0701, NGS 11891-A

PB-33 p. 51; #10 NAA, NAA judd_ngs_0700, NGS 11895-A

PB-34 p. 51; #7 NAA, NAA judd_ngs_0703, NGS 32635-A

PB-35 p. 200; #19 CCNHP Museun Collection, NGS negative #11892, slide 52

PB-36 p. 75; #15 MARI, MARI PS 466, HEE P 312

PB-37 p. 124; #19 NAA, CRA access. #001284, NAA pl14_nm_226

PB-38 p. 37; #20 CCNHP Museum Collection, Chaco Archive A0025235

PB-39 p. 6; #7 AMNH, AMNH access. #000920, HEE 226

PB-40 p. 71; #12 CCNHP Museum Collection, Chaco Archive A0079951, HEE 394

PB-41 p. 87; #15 NAA, NAA judd_ngs_0846, NGS 11866-A

PB-42 p. 90; #14 NAA, NAA judd_ngs_0829, NGS 39555-A

PB-43 p. 87; #7 NAA, NAA judd_ngs_0852, NGS 7658-A

PB-44 p. 180; #11 CCNHP Museum Collection, CHCU PS-4, neg. #78816, CHCU 52336

PB-45 p. 24; #6 CCNHP Museum Collection, Chaco Archive A0059791

PB-46 p. 8; #20 AMNH, AMNH accession #000923, HEE Granger 082

PB-47 p. 21; #1 AMNH, AMNH 2A6461, HEE 647

PB-48 p. 71; #14 CCNHP Museum Collection, Chaco Archive A0079949, HEE 390

PB-49 p. 82; #11 NAA, NAA judd_ngs_0728, NGS 3154

PB-50 p. 90; #3 NAA, NAA judd_ngs_0834, NGS 11875-A

PB-51 p. 205; #2 CCNHP Museum Collection, NGS negative #3206, slide 13

PB-52 p. 21; #5 AMNH, AMNH 2A6464, HEE 650

PB-53 p. 90; #12 NAA, NAA judd_ngs_0832, NGS 11956-A

PB-54 p. 102; #4 CCNHP Museum Collection, Chaco Archive A0079793, HEE 396

PB-55 p. 3; #8 AMNH, AMNH access. #000923, HEE Granger 086

PB-56 p. 11; #4 AMNH, AMNH accession #000923, HEE Granger 080

PB-57 p. 208; #8 AMNH, AMNH 2320_19A

PB-58 p. 53; #16 NAA, NAA judd_ngs_0538, NGS 23232-A

PB-59 p. 105; #16 CCNHP Museum Collection, Chaco Archive A0079895, HEE 306

PB-60 p. 148; #8 NAA, CRA access. #001284, NAA pl14_nm_174

PB-61 p. 27; #4 CCNHP Museum Collection, Chaco Archive A0025394

PB-62 p. 29; #10 CCNHP Museum Collection, Chaco Archive A0030755

PB-63 p. 29; #11 CCNHP Museum Collection, Chaco Archive A0030754

PB-64 p. 197; #10 NAA, NAA PB 4851_cc_087

PB-65 p. 203; #3 CCNHP Museum Collection, NGS negative #32364, slide 79

PB-66 p. 96; #13 NAA, NAA judd_ngs_0816, NGS 39576-A

PB-67 p. 106; #5 NAA, NAA judd_ngs_0807, NGS 34477-A

PB-68 p. 200; #18 CCNHP Museum Collection, NGS negative #28481, slide 27

PB-69 p. 89; #5 NAA, NAA judd_ngs_0841, NGS 11880-A

PB-70 p. 107; #20 CCNHP Museum Collection, Chaco Archive A0079799, HEE 403

PB-71 p. 8; #17 NAA, CRA access. # 001284, NAA judd_mindeleff_043, NAA 3191

PB-72 p. 16; #4 AMNH, AMNH 2A6400, HEE 510

PB-73 p. 21; #2 CCNHP Museum Collection, Chaco Archive A0035372, 29SJ 387-PB

PB-74 p. 22; #16 CCNHP Museum Collection, Chaco Archive A0081467

PB-75 p. 88; #20 NAA, NAA judd_ngs_0686, NGS 22610A

PB-76 p. 117; #7 NAA, NAA judd_nm_037, NAA 239560-A

PB-77 p. 125; #7 NAA, NAA judd_ngs_0336, NGS 39559-A

PB-78 p. 14; #4 NAA, CRA accession #001284, NAA pl14_nm_182

PB-79 p. 15; #16 NAA, NAA judd_ngs_0727, NGS 232324

PB-80 p. 105; #10 NAA, NAA judd_mindeleff_046, NAA 3214

PB-81 p. 16; #1 AMNH, AMNH 2A6398, HEE 506

PB-82 p. 39; #6 CCNHP Museum Collection, Chaco Archive A0025196

PB-83 p. 73; #18 Maxwell Museum of Anthropology, MAX 88_43_177

PB-84 p. 97; #2 NAA, CRA access. # 001284, NAA judd_mindeleff_045, NAA 3209

PB-85 p. 79; #13 NAA, NAA judd_ngs_0962, NGS 285452-A

PB-86 p. 85; #7 NAA, NAA judd_ngs_0866, NGS 15867-A

PB-87 p. 33; #17 AMNH, AMNH 412056, HEE 370

PB-88 p. 85; #4 NAA, NAA judd_ngs_0869, NGS 39595-A

PB-89 p. 86; #14 NAA, NAA judd_ngs_0857, NGS 39553-A

PB-90 p. 86; #2 NAA, NAA judd_ngs_0859, NGS 11933-A

PB-91 p. 4; #7 NAA, NAA judd_ngs_0840, NGS 7645-A

PB-92 p. 21; #19 AMNH, AMNH 12339, HEE 192

PB-93 p. 35; #6 AMNH, AMNH 412083, HEE 357

PB-94 p. 5; #13 AMNH, AMNH accession #000921, HEE 245

PB-95 p. 34; #15 AMNH, AMNH 412072, HEE 345

PB-96 p. 100; #4 CCNHP Museum Collection, Chaco Archive A0079938, HEE 365

PB-97 p. 100 #18 CCNHP Museum Collection, Chaco Archive A0079937, HEE 364

PB-98 p. 19; #13 AMNH, AMNH 2A6460, HEE 646

PB-99 p. 29; #13 AMNH, AMNH 411974, HEE 223

PB-100 p. 200; #11 CCNHP Museum Collection, NGS #18635, slide 26

PB-101 p. 32; #10 AMNH, AMNH 412029, HEE 303

PB-102 p. 97; #19 CCNHP Museum Collection, Chaco Archive A0079943, HEE 371

PB-103 p. 131; #18 NAA, NAA judd_ngs_0626, NGS 22683-A

PB-104 p. 132; #2 NAA, NAA judd_ngs_0642, NGS 22689-A

PB-105 p. 132; #13 NAA, NAA judd_ngs_0547, NGS 22674-A

PB-106 p. 79; #12 NAA, NAA judd_ngs_0964, NGS 28478-A

PB-107 p. 83; #6 NAA, NAA judd_ngs_0887, NGS 22661-A

PB-108 p. 6; #9 AMNH, AMNH accession #000920, HEE 216

PB-109 p. 27; #6 AMNH, AMNH 411936, HEE 167

PB-110 p. 80; #5 NAA, NAA judd_ngs_0895-0897, NGS 28449-A

PB-111 p. 84; #3 NAA, NAA judd_ngs_0878, NGS 18670-A

PB-112 p. 15; #12 NAA, NAA judd_ngs_0706, NGS 28450-A

PB-113 p. 23; #2 CCNHP Museum Collection, Chaco Archive A0081459, MNM 116.56

PB-114 p. 22; #20 CCNHP Museum Collection, Chaco Archive A0081461

PB-115 p. 22; #14 CCNHP Museum Collection, Chaco Archive A0081468

PB-116 p. 125; #3 NAA, NAA judd_ngs_0706, NGS 28486-A

PB-117 p. 81; #6 NAA, NAA judd_ngs_0924, NGS 23341-A

PB-118 p. 84; #4 NAA, NAA judd_ngs_0879, NGS 18622-A

PB-119 p. 96; #6 AMNH, AMNH 125351, HEE 75, 377

PB-120 p. 119; #14 AMNH, AMNH 2320_16B

PB-121 p. 148; #15 NAA, CRA accession #001284, NAA pl14_nm_158

PB-122 p. 201; #10 CCNHP Museum Collection, NGS Negative #34477, slide 40

PB-123 p. 201; #9 CCNHP Mus. Coll., NM-Bonito-plan, 2^nd^ and 1^st^ type rooms, slide 47

PB-124 p. 202; #2 CCNHP Mus. Coll., NM – Bonito – ground plan all types, slide 55

PB-125 p. 119; #5 AMNH, AMNH 2320_18A

PB-126 p. 200; #15 CCNHP Museum Collection, NGS negative #18639, slide 25

PB-127 p. 200; #17 CCNHP Museum Collection, NGS negative #39525A, slide 28

PB-128 p. 137; #14 NAA, NAA judd_ngs_0582, NGS 11925-A

PB-129 p. 202; #6 CCNHP Museum Collection, NGS Negative #4304B, slide 59

PB-130 p. 203; #17 CCNHP Museum Collection, NGS Negative #18643, slide 92

PB-131 p. 188; #19 AMNH, AMNH 411865, HEE 077

PB-132 p. 201; #6 CCNHP Museum Collection, NM- Bonito-R-175, slide 35

PB-133 p. 26; #1 AMNH, AMNH 411903, HEE 126A

PB-134 p. 116; #12 AMNH, AMNH accession #000923, HEE Granger 090

PB-135 p. 14; #1 NAA , CRA accession #001284, NAA pl14_nm_175

PB-136 p. 95; #13 NAA, NAA judd_ngs_0696, NGS 31996-A

PB-137 p. 3; #5 NAA, accession #001113, NAA judd_nm_046
